# Supplementary material for: An evaluation of genetic causes and environmental risks for bilateral optic atrophy
Source: PLoS One. 2019 Nov 25;14(11):e0225656. doi: 10.1371/journal.pone.0225656 (PMC6876833; doi:10.1371/journal.pone.0225656)
Supplement: Attachment — Submitted filename: Response to Reviewers.docx [file pone.0225656.s002.docx]

Obul Reddy Bandapalli, MSc, PhD
Academic Editor
PLOS ONE

Dear Dr. Bandapalli:

Thank you for considering our manuscript and allowing us to address the issues brought forth.

The manuscript has been edited to meet PLOS ONE’s style requirements. In particular, the “References” heading has been changed to the appropriate heading style 1. A caption for “Supporting Information” has been added after the references section. Table 1 has been correctly formatted to have no empty rows with a clearer presentation of information.

The ethics statement has been updated in the manuscript and in the online submission form: “The Hamilton Integrated Research Ethics Board approved the retrieval of data with knowledge of the identifying information.” In addition, any potential link to the patients have been removed – date of birth and specific age of onset have been removed from the S1 Table.

“Fig 1: Flowchart of the retrospective study” has been added to the manuscript to allow readers to understand the process of patient and variant selection for NGS. All in-text citations have been changed accordingly.

Further, we would like to address the comments from the editors in the order in which they were presented.

Sincerely,

Mark

Mark Tarnopolsky, MD, PhD,

Department of Pediatrics, McMaster University,

Hamilton, Ontario, L8N 3Z5, CANADA.

Phone 905-521-2100 (76593)

FAX 905-577-8380

[tarnopol@mcmaster.ca](mailto:tarnopol@mcmaster.ca)

Reviewer # 1:

We would like to thank this reviewer for his/her helpful comments and have made revisions along the lines suggested.

Specifically: We have changed “epigenetic factors” in the abstract and in our hypothesis to “environmental [risk] factors.” In addition to the changes in our hypothesis, we suggest in our discussion that environmental toxins such as chronic ethanol consumption can be an independent cause for the development of OA. With these changes in mind, we believe it is more clear that chronic ethanol consumption is an environmental factor that could be triggering epigenetic changes in known or unknown associated genes, rather than a definitive epigenetic factor for OA. Furthermore, statements have been added to clarify how patients were selected for mtDNA and/or nuclear NGS (lines 117-119). Lastly, a statement has been added to clarify the selection of genes in the PEO/Optic Atrophy nuclear panel (lines 144-146).

Reviewer # 2:

We would like to thank this reviewer for his/her helpful comments and have made revisions along the lines suggested.

Specifically: A pictorial methodology (Fig 1) has been added to describe the retrospective design, criteria, and variant classifications. A sentence on the potential role of DNA repair genes has been added (lines 86-87). The lines 84-90 in the unrevised manuscript has been split into two concise sentences. We have added a sentence to state how the panel was created and that all detected nuclear variants were reported. Therefore, no variants were discovered in *SLC25A1* or the unknown regions/unannotated. The typo found on line 204 in the unrevised manuscript has been fixed. The possibility of a metabolic syndrome cannot be ruled out through our study, as stated in lines 88-91. All patients are tested for B12/folate deficiencies and other causes for OA, prior to NGS. The pictorial methodology added can help to clarify this. Lastly, machine learning heuristics may be an answer in the future for overcoming challenges in sampling, but it may be out of the scope of the current study.
